# The role of Zn ions in the interaction between SARS-CoV-2 orf7a protein and BST2/tetherin

**DOI:** 10.1140/epjp/s13360-023-03731-w

**Published:** 2023-03-08

**Authors:** S. Botticelli, R. Chiaraluce, V. Consalvi, G. La Penna, A. Pasquo, M. Petrosino, O. Proux, G. C. Rossi, F. Stellato, S. Morante

**Affiliations:** 1 Università di Roma “Tor Vergata” and INFN, Sezione di Roma 2 - Via della Ricerca Scientifica 1, 00133 Rome, Italy; 2 Dipartimento di Biochimica, Sapienza Universitá di Roma, Piazzale Aldo Moro 5, 00185 Rome, Italy; 3 CNR, Institute for Chemistry of Organometallic Compounds, 50019 Sesto Fiorentino, Italy; 4 ENEA CR Frascati, Diagnostics and Metrology Laboratory FSN-TECFIS-DIM, Via E. Fermi, 45, 00044 Frascati, Italy; 5 Observatoire des Sciences de l’Univers de Grenoble, UMS 832 CNRS, Université Grenoble Alpes, 38041 Grenoble, France; 6 Centro Fermi - Museo Storico della Fisica e Centro Studi e Ricerche Enrico Fermi, Via Panisperna 89a, 00184 Roma, Italy

## Abstract

In this paper, we provide evidence that Zn$^{2+}$ ions play a role in the SARS-CoV-2 virus strategy to escape the immune response mediated by the BST2-tetherin host protein. This conclusion is based on sequence analysis and molecular dynamics simulations as well as X-ray absorption experiments [1].

## Introduction

An efficient strategy used by living organisms to block virus infections is carried out by proteins belonging to the tetherin family. They are known as BST2 (bone marrow stromal antigen 2) or CD317 (cluster of differentiation 317). BST2 is expressed in many cells, and it is found to be able to counteract viral replication by first trapping enveloped viral progeny on the surface of infected cells, successively leading to virus internalization and degradation.

**Fig. 1:**
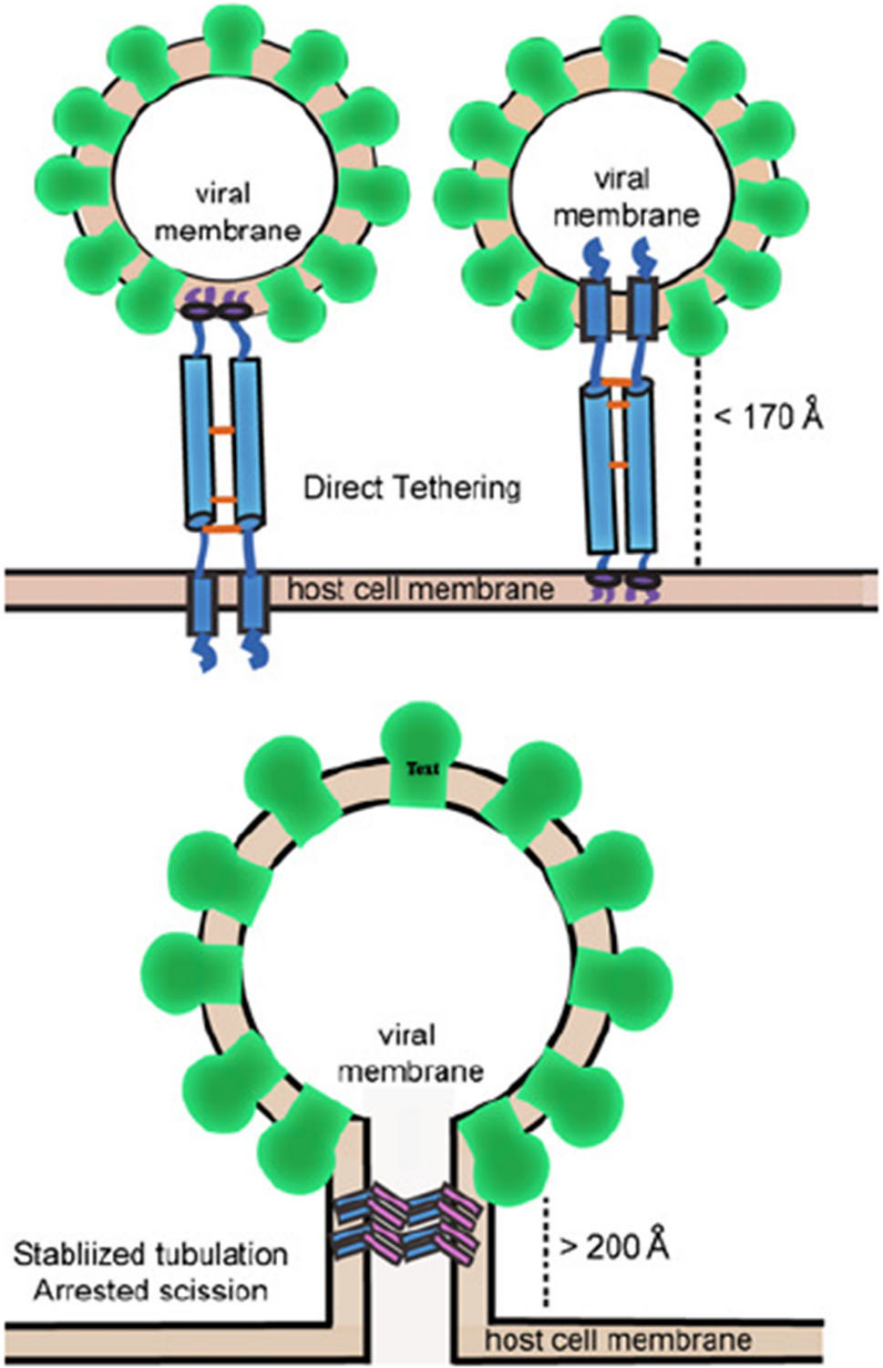
Upper panel: a sketch of the *“direct tethering model”* acted by two BST2 dimers. Lower panel: membrane scission is prevented by bar-like BST2 oligomers. Figure is taken from [2]

The starting point of our investigation is the analysis carried out in Ref. [2] in which two distinct defense mechanisms acted by BST2 have been identified. They are schematically shown in Fig. 1. In the upper part of the figure, BST2 is shown to act by trapping the virus on the surface of the host cell. In the lower part BST2 works as a gate that, upon closing, prevents virus cell inclusion.

**Fig. 2:**
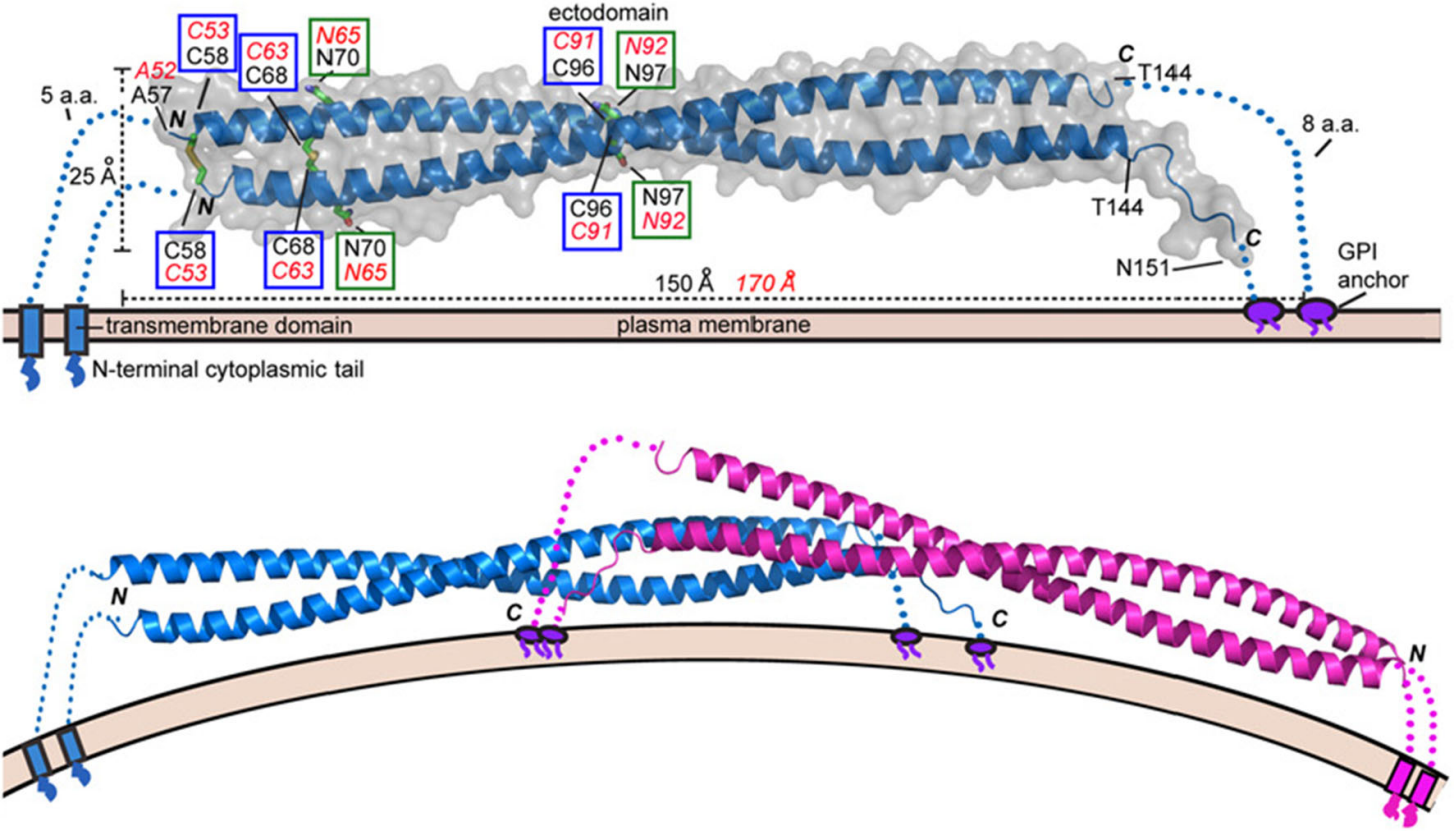
Upper panel: crystal structure of mouse BST2 ectodomain. Disulfide bonds are highlighted in blue boxes. Lower panel: a possible dimeric structure. Figure is taken from [2]

BST2 is a homodimer (see Fig. 2) made by two intertwined alpha-helices. As highlighted in the upper part of Fig. 2, the dimeric structure is stabilized by the formation of covalent intermolecular disulfide bonds between pairs of homologous cysteine (Cys) residues belonging to the two monomers. In detail, three disulfide bonds are formed by the homologous pairs Cys$_{53}$, Cys$_{63}$, and Cys$_{91}$. Note that also the tetrameric structure shown in the bottom panel is stabilized by the formation of inter-molecular disulfide bridges.

**Fig. 3:**
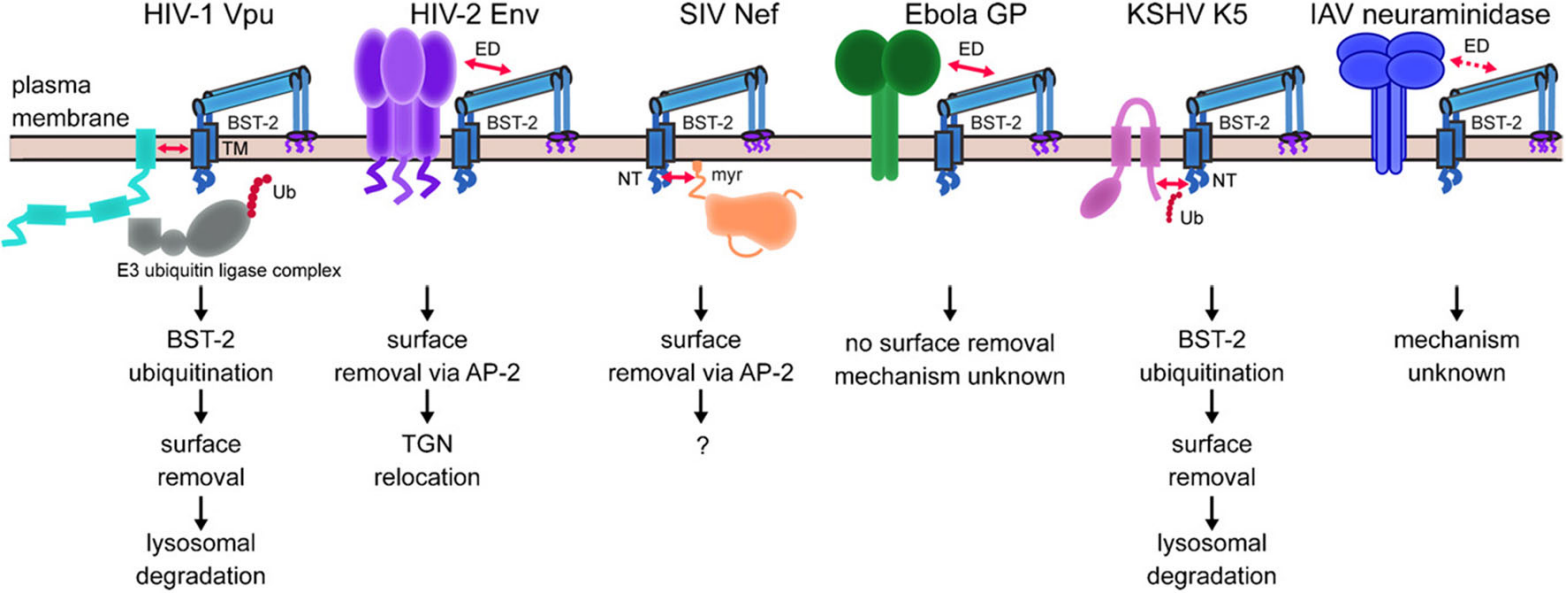
A list of viral proteins known to be able to block the BST2 antiviral action. The figure is taken from [2]

Figure 3 (taken again from Ref. [2]) is a kind of summary of the identified antagonizing anti-BST2 actions known to involve specific viral proteins. This is to illustrate the diversified viral abilities to evade BST2 antiviral action.

## The role of the SARS-CoV-2 orf7a accessory protein

Inspired by the analysis of Ref. [2], we made the hypothesis that orf7a, an accessory protein that is also present in SARS-CoV where it is known to interact with many host proteins, acts in the case of SARS-CoV-2 as a BST2 antagonist. The important novelty we introduce in the present investigation is the idea that orf7a counteracting action is mediated by Zn$^{2+}$ ions. The idea that Zn may play a role comes from two related observations [3].

**Fig. 4:**
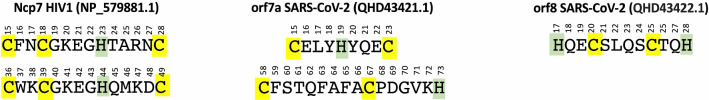
Zn Finger motifs in NP579881 (Ncp7 of HIV-1) (left), QHD43421.1 (orf7a of SARS-CoV-2) (center) and QHD43422.1 (orf8 of SARS-CoV-2) (right). Cys are highlighted in yellow and His in green. Figure is taken from [3]

1. It is known that “*Zn finger motifs*”, i.e., short regions characterized by the close presence along the amino acidic sequence of histidine and cysteine residues occur along certain viral sequences. A notable example (see Fig. 4) is the *Nucleocapsid protein 7* sequence, a key HIV protein that was recognized as a potential target for an effective next-generation antiretroviral therapy [4, 5].
2. We remark that also along the amino acidic sequence of orf7a and orf8 (two SARS-CoV-2 accessory proteins whose specific roles need to be better elucidated), there are a few *Zn finger motifs*. As the name suggests, *Zn finger motifs* are known to be privileged sites for metal ion binding, in particular for Zn$^{2+}$ ions.

### Molecular dynamics, MD

The first step necessary to test and possibly validate our hypothesis is to build a model structure for orf7a^1^ capable of hosting Zn$^{2+}$. To this purpose, we performed MD simulations of the orf7a plus Zn$^{2+}$ complex according to the following protocol [3].

1. We have produced thousands of orf7a chain configurations generated as self-avoiding random walks obtained by random choices of the protein residues dihedral angles (there are 445 dihedral angles involving non-H atoms along the orf7a protein).
2. We monitored and measured the distances between all the pairs of cysteine sulfur atoms *d*(CysCys), as well as between all the pairs having a cysteine sulfur and a histidine nitrogen *d*(CysHis).
3. Two structurally interesting configurations with the *d*(CysCys) and *d*(CysHis) distances smaller than 1.2 nm were found
  one involving the triplet Cys$_{15}\ldots$ His$_{19}\ldots$ Cys$_{23}$
  and a second one the triplet Cys$_{58}\ldots$ Cys$_{67}\ldots$ His$_{73}$
4. We then proceeded to insert a Zn$^{2+}$ ion in both sites
5. MD and energy minimization within a density-functional tight-binding approximation were used to relax the two structures.

**Fig. 5:**
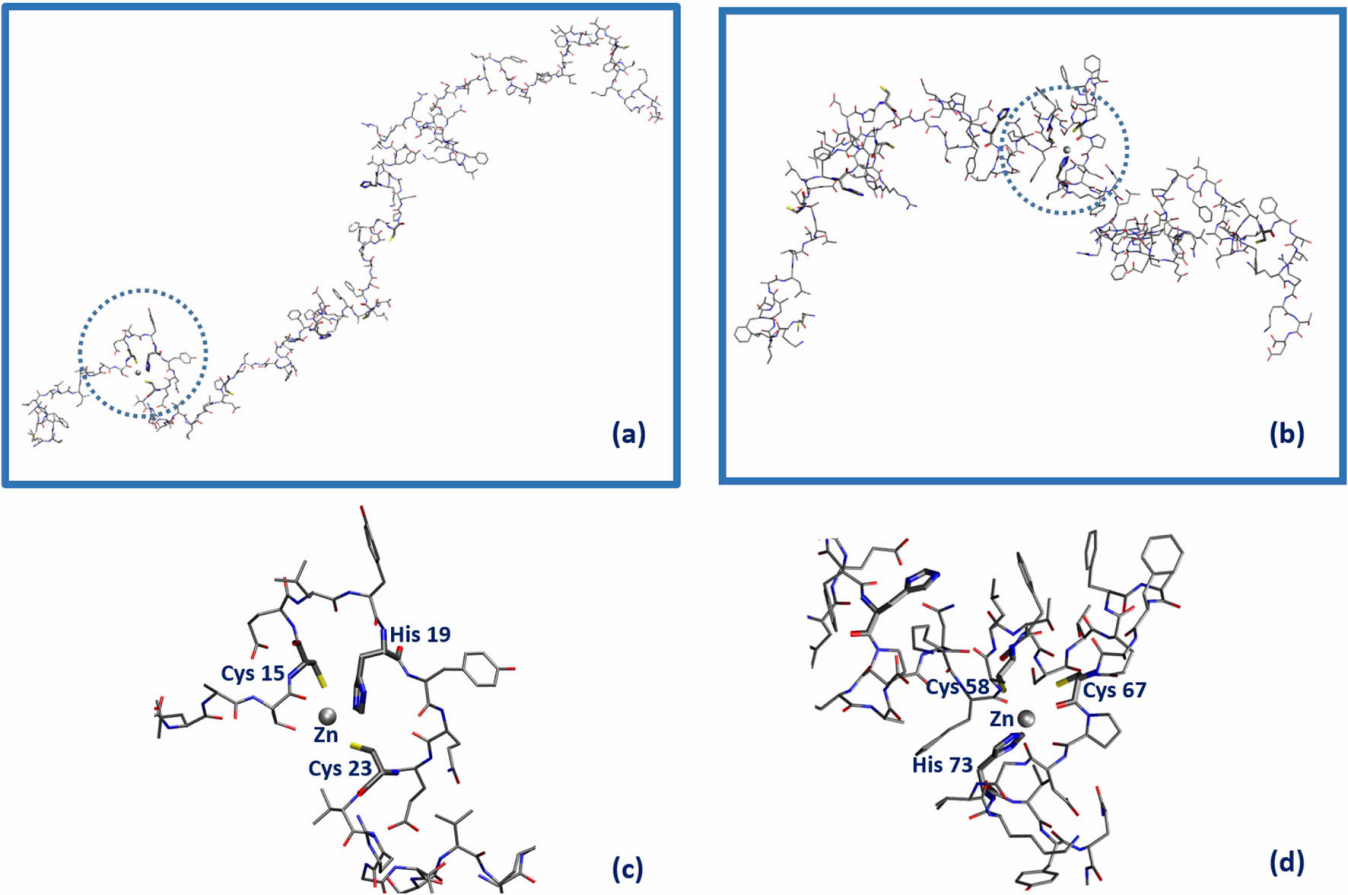
Sketch of the orf7a domains capable of hosting a Zn$^{2+}$ ion (gray sphere). Amino acid residues within a distance below 2.2 Å from Zn are highlighted in bold. Panels (**c**) and (**d**) are the blow-up of the two possible Zn$^{2+}$ binding sites Cys(15)–His(19)–Cys(23) (circled in panel (**a**)) and Cys(58)–Cys(67)–His(73) [circled in panel (**b**)], respectively. Figure is taken from [3]

The simulation strategy described above allowed us to identify two Zn accessible sites along the orf7a chain. They are shown in Fig. 5. In panel (a) the Zn ion is located in the region of the first Zn finger motif (aa: 15–23) and in panel (b) in the region (from aa. 58 to 73) of the second Zn finger motif. Panels (c) and (d) show blow-ups of the two Zn binding regions we have identified.

The next step would be to include BST2 in the game to study how the latter can interact with the orf7a/Zn complex. This will be the object of a forthcoming investigation [6], where we want to check the idea that the Zn assisted orf7a/BST2 interaction can lead to the formation of a crossed disulfide bond between orf7a and BST2, thus disrupting the BST2 dimeric (or tetrameric) structure and consequently blocking its antiviral action.

### X-ray absorption spectroscopy

In order to give an experimental basis to the interesting scenario described above we have performed a detailed X-ray absorption spectroscopy (XAS) study of the various (protein/metal and protein/protein/metal) complexes of relevance here (see below Table 1)

X-ray absorption spectroscopy (XAS) is the experimental technique of election when dealing with heavy atoms (like metals) in interaction with proteins in solution. XAS is a powerful tool that allows extracting information at atomic resolution about the way metal ions bind biological stuff even at low concentrations like the ones we encounter in actual biological samples.

The reason is that ion binding to histidine and to a lesser extent to cysteine residues leaves unmistakable features in the XAS spectrum of the sample. In fact, Zn is bound to His residue through a nitrogen belonging to the rigid imidazole ring. Multiple scattering processes enhance the His contribution making it easily recognizable. For what concerns Cys, the metal ion is bound via a sulfur atom. The latter sticks out as it is much heavier than all the lighter carbon, oxygen and nitrogen ligands that are the leading atoms of the side chain of the other nearby amino acid residues.

In order to decide whether Zn is involved in virus anti-BST2 strategy, we need to answer the following questions:

1. Is Zn interacting with the viral accessory proteins, orf7a, and/or with BST2?
2. If the answer to this question is positive, we must address two further issues.
  If, to start with, Zn is bound to orf7a, is the orf7a/metal complex able to interact with BST2?
  Can this interaction possibly alter or (even) impair the BST2 antiviral activity?

A careful analysis of the XAS data collected for the samples listed in Table 1 can help settling the questions above. In the table, we provide a brief description of the samples we have subjected to XAS measurements.

**Table 1:** List of samples for which XAS data were acquired, with the indication of protein and Zn concentration in mM

	Sample name	Color	[BST2]	[orf7a$_{\mathrm{L1}}$]	[orf7a$_{\mathrm{L2}}$]	[Zn]
1	BST2	Blue	0.11	–	–	0.1
2	orf7a$_{\mathrm{L1}}$	Red	–	0.03	–	0.027
3	orf7a$_{\mathrm{L2}}$	Green	–	–	0.03	0.027
4	BST2$_{\mathrm{first}}$+orf7a$_{\mathrm{L1}}$	Purple	0.11	0.03	–	0.1
5	BST2 + orf7a$_{\mathrm{{L2}}\,\mathrm{{first}}}$	Orange	0.11	–	0.03	0.1
6	Zn-buffer	Grey	–	–	–	2

In the second column, we report the name of the sample; in the third, the color we will adopt to report the XAS data in the following figures; in the fourth, fifth and sixth, we give the concentration of the various proteins present in each sample where it applies, in the last column the Zn concentration.

The labels L1 and L2 associated with the name of the orf7a protein (rows 2 and 3) have been introduced to distinguish between two orf7a constructs differing by the absence ($\text {orf7a}_{\mathrm{L1}}$) or the presence ($\text {orf7a}_{\mathrm{L2}}$) of $\text {Cys}_{15}$ at the beginning of the protein amino acid sequence. The subscript first appearing next to BST2 and orf7a$_{\mathrm{L2}}$ in the fourth and fifth row, respectively, are there to recall that in the case of the sample $\text {BST2}_{\text {first}}+\text {orf7a}_{\mathrm{L1}}$, the $\text {orf7a}_{\mathrm{L1}}$ protein was added to an already Zn-loaded BST2 solution, while, vice versa in the case of the sample BST2+orf7a$_{\mathrm{L2 first}}$, BST2 was added to an already Zn-loaded orf7a$_{\mathrm{L2}}$ solution. Note that the Zn concentration has been always taken to be sub-stoichiometric in order to minimize possible spurious XAS signals from free Zn in solution. In row 6, the concentration of Zn in buffer is reported.

**Fig. 6:**
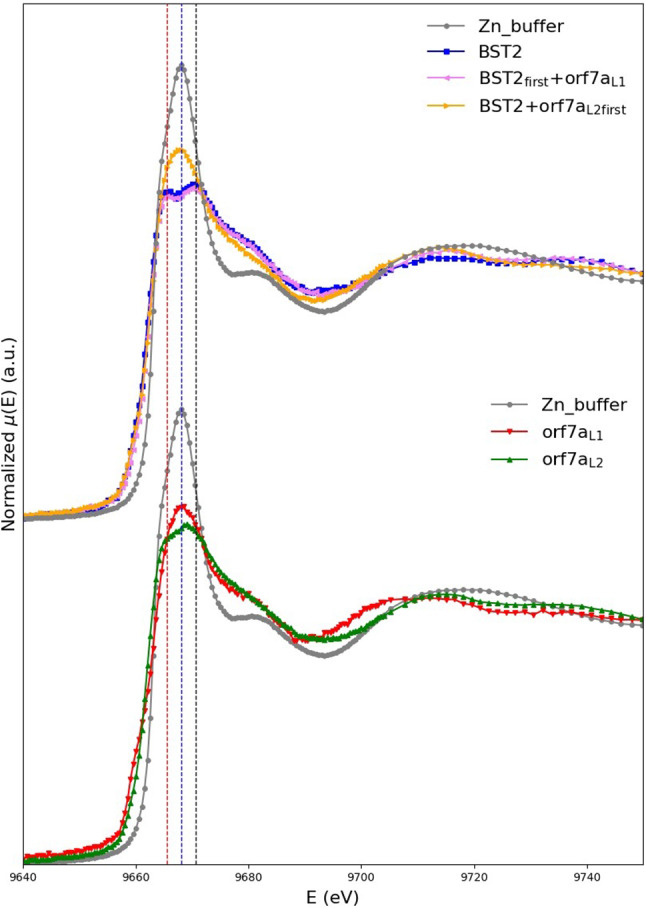
In the top panel, we compare the normalized XANES absorption coefficient of the Zn-buffer (grey curve) to that of BST2 (blue curve), $\text {BST2}_{\mathrm{first}}+\text {orf7a}_{\mathrm{L1}}$ (purple curve) and $\text {BST2}+\text {orf7a}_{\mathrm{{L2}}\,\textrm{first}}$ (orange curve). In the bottom panel, we compare the normalized XANES absorption coefficient of the Zn-buffer (grey curve) to that of $\text {orf7a}_{\mathrm{L1}}$ (red curve) and $\text {orf7a}_{\mathrm{L2}}$ (green curve). The central dotted line is drawn to pass through the maximum of the Zn buffer spectrum at 9668.1 eV, and the other two through the positions of the split maxima of the BST2 spectrum, located at 9665.6 and 9670.6 eV, respectively. The figure is taken from [1]

In Fig. 6, we summarize and compare the most significant among the measured X-ray spectra. In this work, we limit the discussion to the XANES region of the spectrum.

From a general analysis of these data, one can get a number of interesting information. First of all, we may remark that the Zn buffer spectrum (grey) is significantly different both from BST2 spectrum (blue curve in the top panel) and from the spectra of orf7a (green and red curves in the bottom panel) irrespective of the presence of Cys$_{15}$. This means that all the three systems are able to bind Zn (or in other words that not all the Zn is free in solution as it is the case in the buffer).

More in detail, we notice that orf7a$_{\mathrm{L1}}$ and orf7a$_{\mathrm{L2}}$, whose sequences only differ for the presence of the initial Cys$_{15}$ residue in orf7a$_{\mathrm{L2}}$, show different XANES spectral features, meaning that this extra cysteine is involved in Zn binding.

But the most interesting observation is that the orange spectrum, which corresponds to a sample where Zn was already bound to orf7a$_{\mathrm{L2}}$ when BST2 was added to the solution, is different from the orf7a$_{\mathrm{L2}}$ spectrum (green curve) thus suggesting that somehow BST2 interferes with the orf7a$_{\mathrm{L2}}$-Zn binding.

Finally, it is worth noticing that the double peak feature in the white line is commonly interpreted as the simultaneous presence of Cys and His as metal ligands. This fact was also confirmed by the first principle XANES calculations that we have expressly performed and reported in [1].

### Conclusions

The work we have summarized in this talk is a first step in the direction of understanding the possible role of Zn in the interaction between BST2 and some of the accessory SARS-CoV-2 proteins. Experimental XAS data supported by first principle XANES calculations demonstrate that both the host BST2 protein and the viral orf7a protein are able to bind Zn. Furthermore, a detailed comparison of the spectra of the samples of Table 1 supports the conjecture that BST2/orf7a complexes get formed. The possible consequent disruption of the BST2 functional polymerization may be at the basis of the inactivation of the BST2 antiviral activity.

There are, of course, many other questions we would like to answer and work is in progress both from a numerical and experimental point of view.

It goes without saying that once (if ever) our hypothesis will be demonstrated to be correct, it would be an important step toward a better understanding of the strategies devised by coronaviruses to circumvent our immunological defenses and possibly suggest some therapeutical strategy.

## Data Availability

The datasets generated during and/or analyzed during the current study are available from the corresponding author on reasonable request.

## Footnotes

1 orf7a belongs to the class of IDP (Intrinsically Disordered Proteins), thus the crystal structure of the whole protein is not available.
